# Practical strategies for handling breakdown of multiple imputation procedures

**DOI:** 10.1186/s12982-021-00095-3

**Published:** 2021-04-01

**Authors:** Cattram D. Nguyen, John B. Carlin, Katherine J. Lee

**Affiliations:** 1 Clinical Epidemiology and Biostatistics Unit, Murdoch Children’s Research Institute, The Royal Children’s Hospital, Flemington Road, Parkville, Victoria 3052 Australia; 2 Department of Paediatrics, Faculty of Medicine, Dentistry and Health Sciences, University of Melbourne, The Royal Children’s Hospital, Flemington Road, Parkville, Victoria 3052 Australia

**Keywords:** Auxiliary variables, Collinearity, Convergence, Missing data, Multiple imputation, Multivariate imputation by chained equations, Multivariate normal imputation, Perfect prediction

## Abstract

Multiple imputation is a recommended method for handling incomplete data problems. One of the barriers to its successful use is the breakdown of the multiple imputation procedure, often due to numerical problems with the algorithms used within the imputation process. These problems frequently occur when imputation models contain large numbers of variables, especially with the popular approach of multivariate imputation by chained equations. This paper describes common causes of failure of the imputation procedure including perfect prediction and collinearity, focusing on issues when using Stata software. We outline a number of strategies for addressing these issues, including imputation of composite variables instead of individual components, introducing prior information and changing the form of the imputation model. These strategies are illustrated using a case study based on data from the Longitudinal Study of Australian Children.

**Supplementary Information:**

The online version contains supplementary material available at 10.1186/s12982-021-00095-3.

## Background

Multiple imputation (MI) is a popular method for handling missing data. The missing data are replaced with multiple ($m > 1$) imputed values to produce $m$ completed datasets. Standard analysis methods are applied to each of the $m$ completed datasets, and the resulting estimates for quantities of interest are combined using Rubin’s rules [1].

There are many methods for generating imputed data, most of which rely on complex algorithms [2]. There are two predominant methods for imputing missing data in multiple variables. The first of these, multivariate normal imputation (MVNI), assumes that variables requiring imputation follow a joint multivariate normal distribution [2]. MVNI is implemented using the data augmentation (DA) algorithm, an iterative procedure that alternates between drawing imputed values for the missing data and drawing values of the imputation model parameters.

The second method is multivariate imputation by chained equations (MICE), also known as fully conditional specification (FCS), which imputes the missing values on a variable-by-variable basis using a series of univariate imputation models, one for each incomplete variable [3, 4]. The univariate models are fitted iteratively, with each variable imputed in turn, conditioning on the completely observed variables and the most recent imputed values of incomplete variables. The algorithm is run multiple times in parallel to obtain $m$ imputed datasets [5]. When using MICE, the univariate imputation models are tailored to the variable being imputed. In particular, generalized linear models are used to impute non-continuous variables, using maximum likelihood estimation (MLE) to fit these models, which also relies on iterative algorithms [6].

Although these two MI procedures are widely available in statistical software [7], barriers to their successful implementation arise from numerical problems that occur within the iterative procedures, which can lead to termination of the procedure without imputed values being generated [8–10]. We refer to these issues as “numerical problems”, “failure” or “breakdown” of the MI algorithms. We avoid the term “model non-convergence” to avoid ambiguity with other types of convergence, particularly the stabilization of iterative procedures to their target distribution. Guidance on assessing the convergence of MI algorithms may be found elsewhere [2, 5].

The aim of this paper is to describe common causes of numerical problems in MI, and to provide practical guidance for handling such problems based on recommendations from the literature and our experience as users of MI. In the next section, we motivate this work with an example from the Longitudinal Study of Australian Children. We then describe perfect prediction and collinearity, two key issues that can lead to failure of the MI procedure. Finally, we present some strategies for diagnosing and overcoming these issues, which we illustrate in a case study. This paper focuses on numerical problems encountered using Stata software, and we provide Stata code as Additional file2.

## Example: Longitudinal Study of Australian Children

For illustrative motivation we use data from the Longitudinal Study of Australian Children (LSAC) Kindergarten cohort, consisting of 4983 children aged 4–5 years recruited in 2004 [11]. Our analysis examined the association between body mass index (BMI) Z-score at 4–5 years of age and health related quality of life (HRQoL) problems at 8–9 years, adopting a simplified version of a published analysis [12].

### Analysis model

The analysis model was a logistic regression of HRQoL problems on BMI Z-scores with adjustment for potential confounders. HRQoL was measured using the PedsQL, a 23 item-scale that asked parents about the frequency of their child’s health-related problems. Responses ranged from 1 to 5, and were reverse scored as follows: 1 = 100, 2 = 75, 3 = 50, 4 = 25, and 5 = 0. The items were averaged to produce a total score (range 0–100), which was dichotomized (at 1 standard deviation above the population mean) to produce a binary variable [13]. The exposure of interest was BMI Z-score, which was derived using direct measurements of weight and height, and standardized by age and sex. The covariates in the logistic regression model were mother’s education, maternal language, child’s indigenous status, child sex, child age in months, mother’s work, neighborhood disadvantage, mother’s psychological distress and child mental health (see Additional file 1: Supplementary Table 1 for further details).

### Missing data

Twenty-four percent (1180/4983) of participants were missing all HRQoL items, while 7% (325/4983) were missing individual items. Only 3039 (61%) participants had completely observed data for all variables required for the analysis. Those with completely observed data were more likely to have English as their main language (90% vs. 81%), were less likely to be indigenous (2% vs. 6%) or be in a sole-parent household (11% vs. 20%), and their mothers had higher rates of school completion (64% vs. 51%) compared with those with incomplete data.

### Imputation model

Having decided to use MI to handle the missing values, all variables in the analysis were included in the imputation model [14, 15]. We imputed the individual HRQoL items and used these to derive the binary outcome variable. We also included HRQoL measurements from earlier waves in the imputation model as they were correlated with the incomplete outcome. In total there were 54 variables in the imputation model: 23 individual HRQoL items (used to derive the outcome variable), 10 covariates from the analysis model and 21 HRQoL items from an earlier wave included as auxiliary variables. MI was initially implemented using MICE in Stata 15 [16], with linear regression for imputation of continuous variables, logistic regression for binary variables and ordinal logistic regression for the HRQoL items. However, the MICE procedure failed and no imputed values were generated.

## What are common causes of numerical problems with imputation algorithms?

In this section, we describe perfect prediction and collinearity, two of the main problems that lead to numerical problems with MI.

### Perfect prediction

When fitting generalized linear models to categorical data, a common cause of numerical problems is perfect prediction [17, 18]. Perfect prediction can occur if a covariate (or combination of covariates) completely discriminates the outcome categories. If this is the case, maximum likelihood estimates may not exist (or lie on the boundary of the parameter space), leading to numerical issues when fitting the imputation model.

To illustrate the issue of perfect prediction, consider the simple missing data example shown in Table 1. This dataset consists of a binary variable Y, and an unordered categorical variable X. There are missing values in both variables, with observed data for both variables in 54/70 (77%) of cases. When we try to impute the missing values in Stata [16] using MICE (with logistic regression to impute Y and multinomial logistic regression to impute X), the imputation procedure fails and Stata produces the message: “variables that perfectly predict an outcome were detected when logit executed on the observed data”. A logistic regression of Y on X leads to problems with perfect prediction, because all cases with X = 2 have the outcome Y = 0.

**Table 1:** Cross-tabulation of two simulated variables Y (binary) and X (categorical)

Y	X				Total
	0	1	2	Missing	
0	25	17	2	6	50
1	6	4	0	1	11
Missing	5	2	1	1	9
Total	36	23	3	8	70

### Collinearity

Collinearity occurs when covariates in the imputation model are highly correlated, leading to difficulties in estimating separate effects for each of the correlated covariates, which manifest in unstable estimates with inflated variances [19], and which can in some cases lead to failure of iterative estimation algorithms.

To illustrate the issue of collinearity, we simulated a dataset (n = 120) with a binary outcome variable (Y) and four variables measured on a 5-point Likert scale (V1–V4). V1–V4 were designed to be highly correlated (Table 2) in order to produce numerical problems due to collinearity. There was 10% missing data in each of V1–V4, which we attempted to impute using MVNI in Stata (where the ordinal variables were each represented by four indicator variables [20]). The MVNI procedure failed without producing any imputed data and the software issued a message that there were “collinear imputation (dependent) variables detected”.

**Table 2:** Correlation matrix of simulated variables Y, V1, V2, V3 and V4

	Y	V1	V2	V3	V4
Y	1				
V1	− 0.09	1			
V2	− 0.02	0.94	1		
V3	− 0.18	0.92	0.92	1	
V4	− 0.18	0.89	0.93	0.96	1

### Challenges to MI algorithms: the large model problem

Problems such as collinearity and perfect prediction are more likely to occur when imputation models contain large numbers of variables (meaning many parameters to estimate) relative to the number of observations. In particular, collinearity can occur when imputing repeated measures of a variable, as this can lead to large imputation models with several highly correlated variables. The probability of perfect prediction also increases as sample size decreases, as the number of dichotomous covariates increases, and the balance of the dichotomous covariates decreases [17].

Although the two toy examples described in the previous sections are simplifications of real data problems, such issues are not uncommon in practice. If following MI guidelines, imputation models should include all variables that appear in subsequent analyses, to ensure that relationships of interest are preserved in the imputed data [14]. The model will also include auxiliary variables that are not of substantive interest, e.g. variables that are correlated with the incomplete variables (such as repeated measures) are included to improve the precision of MI estimates [14, 21]. If MI users adopt an “inclusive” variable selection strategy to avoid the omission of important auxiliary variables [21], then imputation models will contain many more variables than those used for substantive analyses. Given the size and complexity of typical imputation models, it is unsurprising that imputation procedures commonly fail.

### Exploring reasons for the breakdown of the imputation procedure

A useful initial step when imputation procedures fail is to explore the data to investigate possible reasons for the breakdown. Table 3 provides a number of strategies that can be used to investigate and diagnose problems with imputation models.

**Table 3:** Strategies for exploring reasons for failed imputation procedures

Strategy	Problem identified
Remove variables from the imputation model in turn	If the model runs successfully after omitting a particular variable, this might provide some insight into which variable(s) is causing the problem
Create cross-tabulations of categorical variables in the imputation model (such as that shown in Table 1)	Look for sparse or empty cells as these may be causing perfect prediction. It may be necessary to explore patterns across > 2 variables, as perfect prediction can occur for strata produced by combinations of multiple variables
Explore correlations between variables	This can help identify possible sources of collinearity
Examine any output the software produces prior to breakdown of the MI procedure e.g. interim estimates of model parameters	Look for signs of collinearity such as large standard errors and unstable coefficients across iterations. Omission of variables from a model might also signal perfect prediction or collinearity. If the imputation procedure iterates for a substantial amount of time, it might be advisable to run a small number of iterations in order to obtain some output
For problems with MICE, the univariate imputation models can be tested outside the MICE framework by fitting models to observed data (i.e. complete cases)	Check whether the software removes any variables or issues warnings when fitting the univariate models (as these error messages might provide information that is not provided after imputation model failure). When fitting the univariate models, it is also possible to use additional diagnostics such as the variance inflation factor, which provides an indication of whether standard errors are inflated due to collinearity [22]

## Strategies for handling breakdown of the imputation procedure

After exploring reasons for imputation model breakdown, a number of strategies can be attempted to overcome these issues, which we outline below, noting that individual strategies may be more or less useful for a particular problem. Although we have suggested possible modifications to the imputation model, it is important to ensure that the model remains sensible. For example, variables in the substantive analysis should always be retained in the imputation model. It is also important to consider compatibility, i.e. that the imputation model incorporates the same relationships as the analysis model [23, 24]. Further information on imputation model building is available in the literature [5, 10, 14].

### Reduce the number of auxiliary variables in the imputation model

It may be helpful to reduce the size of the imputation model by removing non-essential auxiliary variables, particularly if:

- *They have large amounts of missing data*, particularly if auxiliary variables are missing for the subgroup of incomplete cases [5, 25].
- *They are not associated with the incomplete variables*. If there is a main variable being imputed, one rule of thumb is to include an auxiliary variable if its correlation with the main variable is ≥0.5 (in absolute value) [25].
- *They are highly correlated with other auxiliary variables.* In this case, there might not be added gain in including both/all of the auxiliary variables.

We note, however, that removing auxiliary variables from the imputation model is not necessarily desirable. An alternative to removing variables is to use a dimension reduction technique. For example, Howard et al. [26] suggest performing principal components analysis of the auxiliary variables, and including a small number of components in the imputation model instead of the original variables.

### Impute composite variables instead of individual components

When working with multi-item scales (e.g., HRQoL), where a total score is derived from multiple items, imputation models can become very large if imputing the individual items. If the scales are being used as auxiliary variables, the imputation model can be simplified by including the total scores or subscale scores rather than the individual items [27, 28].

If the total score is the variable of interest (rather than auxiliary variables), then it is also possible to impute the total score directly rather than the individual items. However, recommendations regarding item- and total-level imputation are unclear. Simulation studies have found that imputing total scores directly can produce less precise estimates compared to imputing the individual items, although the two approaches have been found to have similar performance with respect to bias [29, 30]. Rombach et al.[8] reported more problems with model breakdown when imputing at the item level and superior performance of total-level imputation with smaller sample sizes (< 200). In terms of compatibility of the analysis and imputation models, it may be favorable to impute variables in the same form as they will appear in subsequent analysis.

### Reduce problems with perfect prediction

If imputing categorical variables with > 2 categories, one simple approach for handling sparsity is to collapse categories to produce larger cell sizes. This strategy may only apply in situations where collapsing categories is sensible from a substantive perspective, and the original categorization is not required for subsequent analyses.

White et al. [9] proposed a method that augments the dataset with additional “pseudo-observations” to prevent the outcome from being perfectly predicted. This augmentation procedure has been incorporated into the MI functions of popular statistical packages including Stata, R and SAS [3, 16, 31]. White et al. [9] also outline a number of alternative imputation approaches (e.g., bootstrap and penalised regression methods) that can avoid perfect prediction; however, these approaches may not be as convenient as the augmented data procedure. We note, however, that no imputation approach can recover information on rare categories and that this may be a limitation of the data at hand.

### Introduce prior information

The augmented data approach can be regarded as an informal Bayesian method, in that it introduces additional “prior” information to stabilize estimation. More formal Bayesian approaches can also assist in the stabilization of MI algorithms. For example, when using MVNI, covariance matrices may be unreliably estimated with large amounts of missing data or highly correlated variables [2]. A recommended approach for handling these problems is to specify a ridge prior distribution within the DA algorithm, which shrinks estimated correlations between variables towards zero, which can ameliorate problems with numerical instability [2, 16, 32]. Similarly, for problems with perfect prediction, an explicitly Bayesian imputation method with a weakly informative prior distribution may be used (e.g., Student *t* prior distributions on regression coefficients of generalised linear models, which has been implemented in R) [5, 9, 33, 34].

### Change the functional form of the imputation model

Changing the form of the imputation model may also ameliorate numerical problems. For example, if problems occur with MICE, one could change to MVNI. MVNI is generally more robust to numerical problems than MICE as it jointly estimates the mean vector and covariance matrix of the imputation model, compared to a MICE procedure comprising numerous univariate models.

Within MICE, if there are numerical problems using ordinal logistic regression to impute an ordered categorical variable (e.g., due to perfect prediction), an alternative would be to change the form of the imputation model to a linear regression. Another option is to impute using predictive mean matching (PMM) [35]. PMM replaces each missing value with observed values that are borrowed from donors with similar predicted values from a linear regression model. Alternatively, there are imputation methods that assume an underlying continuous latent distribution for categorical variables (which can implemented, for example, using the “jomo” package in R software) [36, 37]. These alternative approaches may be more robust to issues with sparseness of categorical variables compared with logistic regression approaches.

### Impute longitudinal data using specialized methods for longitudinal data

Imputation procedures often fail when imputing longitudinal data, particularly when imputing in “wide” format, where there is one row for each individual, and repeated measurements are treated as separate variables. To reduce the size of the imputation model when imputing in wide format using MICE, it is possible to use “two-fold FCS”. Under this method, a variable at one time-point is imputed conditional only on information from the same time-point and adjacent time-points, thereby reducing the number of variables in each univariate model within MICE [38, 39]. One could also use a more tailored approach for imputing longitudinal data, such as imputing the data in “long” format (where each longitudinal variable is represented by a single variable, with one row for each repeated measurement) using a multilevel imputation model [37, 40, 41]. For an overview of multiple imputation methods for longitudinal data, we refer to Huque et al. [42].

## Application of strategies to the case study

**Fig. 1:**
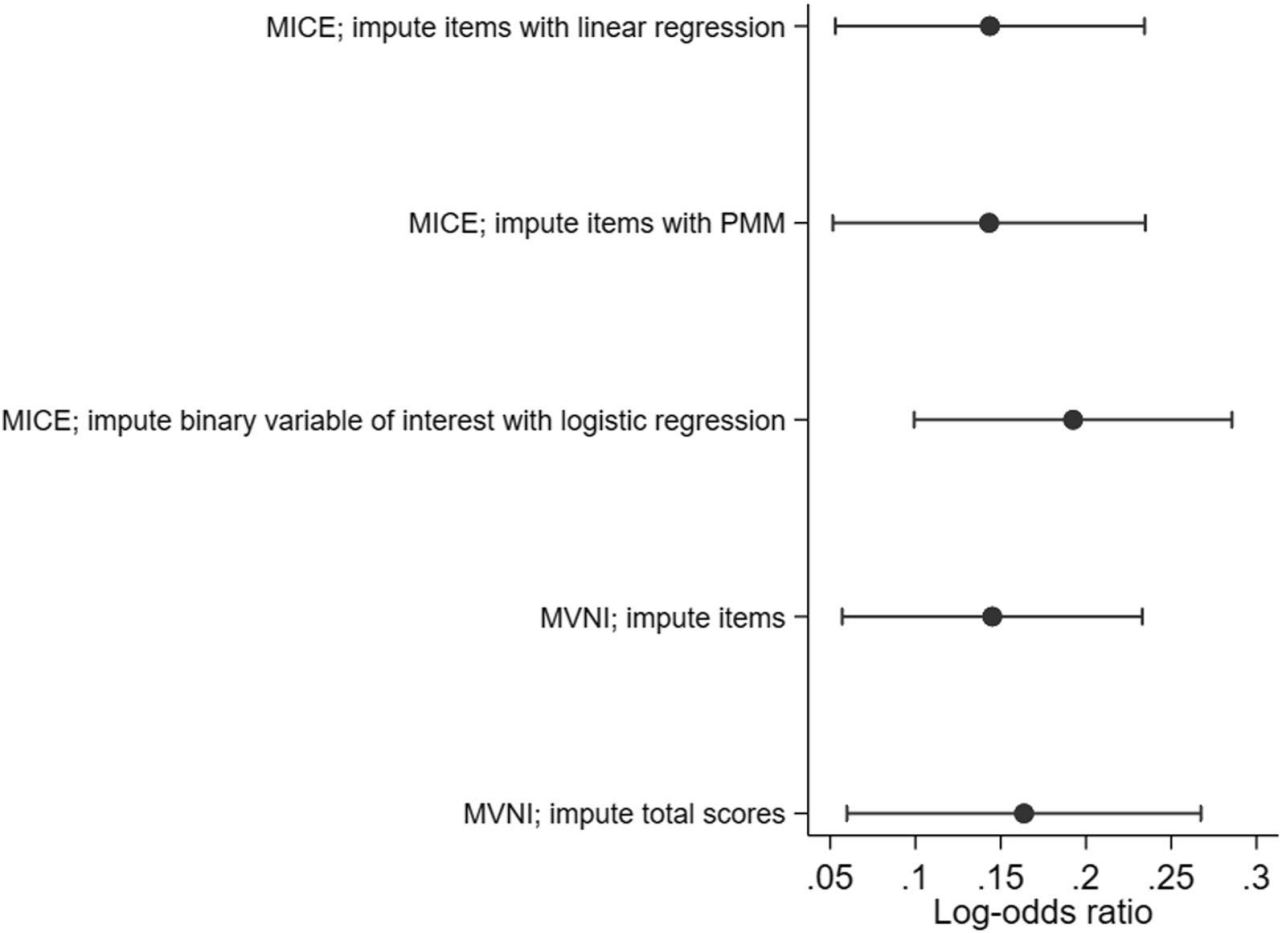
Results of five imputation approaches applied to data from the Longitudinal Study of Australian Children. Results shown are log-odds ratios and 95% confidence intervals for the association between BMI z-score and poor health related quality of life. The five imputation approaches correspond to the scenarios with a “Yes” in the “Successful?” column in Additional file 1: Supplementary Table 2, i.e. strategies 2, 3, 8, 9 and 11 respectively. MICE, multivariate imputation by chained equations; MVNI, multivariate normal imputation

Our case study had a number of challenges that led to numerical problems: multiple correlated items from multiple waves that were being imputed as ordinal categorical variables. We applied several of the strategies described above to the LSAC example, either alone or in combination (see in Additional file 1: Supplementary Table 2). We overcame imputation model breakdown by imputing the binary HRQoL outcome variable directly within MICE, or by imputing the continuous total score using MVNI (and rounding the imputed values for analysis). We were also able to impute the individual HRQoL items using either linear regression or PMM univariate models within MICE (instead of ordinal logistic regression), or by imputing the items using MVNI. Figure 1 shows the estimates for the log-odds ratio of interest for these five approaches. There was some variability in the estimates of the odds ratios, but the overall conclusion was similar, with the odds of HRQoL problems increasing by around 15% per unit of BMI Z-score.

## Conclusions

In this paper, we described common problems that lead to breakdown of imputation algorithms. We also outlined methods for diagnosing the cause of imputation model failure, as well as strategies for overcoming the underlying issues. We demonstrated how these strategies were used to overcome numerical problems in a case study. Although we were able to successfully generate imputations in our case study using a number of the strategies outlined, a limitation with a real data analysis is that it is difficult to know which imputation method is likely to produce the most valid results. In practice it may be useful to perform sensitivity analyses by using a few imputation strategies and examining the robustness of the results as we have done here. A further limitation is that we have focused predominantly on numerical issues encountered when using MI in Stata software, although the issues would be similar in other packages. Some trade-offs are likely to arise when applying the suggested strategies for alleviating numerical problems. For example, removing auxiliary variables or imputing the total scores may enable the imputation model to run, but this might be at the expense of precision of the estimates. In addition, although we have suggested possible modifications to imputation models, we emphasize the importance of considering whether these modifications are sensible [5, 10, 14]. In particular, it is important that the imputation remains compatible with the analysis model. Finally, we recommend that MI users check that iterative imputation procedures have converged/stabilized [2], and also check imputation models as far as possible to ensure that imputed values and the resulting inference(s) are sensible [43, 44].

## Supplementary Information

**Additional file 1: Supplementary Table 1.** Description of variables to be included in analysis model applied to data from the Longitudinal Study of Australian Children (n = 4983). Supplementary **Table 2.** Model specifications for 11 imputation approaches applied to the data from the Longitudinal Study of Australian Children.

**Additional file 2.** Stata syntax for analyses of data from the Longitudinal Study of Australian Children.

## Data Availability

The authors are not authorised to share the data, but access to data can be requested at https://dataverse.ada.edu.au/dataverse/ncld. Stata code for analyses in this paper have been made available as Additional file2.

## Abbreviations

BMI: Body mass index
DA: Data augmentation
FCS: Fully conditional specification
HRQoL: Health related quality of life
LSAC: Longitudinal Study of Australian Children
MI: Multiple imputation
MICE: Multivariate imputation by chained equations
MLE: Maximum likelihood estimation
MVNI: Multivariate normal imputation
