# Supplementary material for: Practical strategies for handling breakdown of multiple imputation procedures
Source: Emerg Themes Epidemiol. 2021 Apr 1;18:5. doi: 10.1186/s12982-021-00095-3 (PMC8017730; doi:10.1186/s12982-021-00095-3)
Supplement: Supplementary file 1 — Additional file 1: Supplementary Table 1. Description of variables to be included in analysis model applied to data from the Longitudinal Study of Australian Children (n = 4983). Supplementary Table 2. Model specifications for 11 imputation approaches applied to the data from the Longitudinal Study of Australian Children. [file 12982_2021_95_MOESM1_ESM.docx]

Supplementary Table 1. Description of variables to be included in analysis model applied to data from the Longitudinal Study of Australian Children (n=4983)

| Variable | Variable type | Detail | Missing data, number (%) |
| --- | --- | --- | --- |
| HRQoL problems | Outcome variable | Binary variable derived from PedsQL (23-item scale)  0= no HRQoL problems, 1 = HRQoL problems | 1505 (30.2) |
| BMI Z-score | Exposure variable | Z-score derived using direct measurements of height and weight, and standardized by age and sex using US Centers for Disease Control growth reference data | 49 (1.0) |
| Mother’s education | Confounder | 0=did not complete high school, 1=completed high school | 44 (0.9) |
| Mother’s language | Confounder | 0 = mother’s main language is English, 1 = mother’s main language is not English | 116 (2.3) |
| Mother’s work status | Confounder | 0 = not working, 1 = working | 48 (1.0) |
| Mother’s emotional distress | Confounder | Kessler-6 score; range 0-24; higher scores indicate more distress | 879 (17.6) |
| Child’s indigenous status | Confounder | 0=non-indigenous, 1=indigenous | 2 (0.04) |
| Child sex | Confounder | 0=female, 1=male | 0 (0) |
| Child’s age | Confounder | Age in months; range 95-119 | 652 (13.1) |
| Child mental health | Confounder | Strengths and Difficulties Questionnaire; range 0-10; higher scores represent worse problems. | 15 (0.3) |
| Neighbourhood disadvantage | Confounder | Socio-Economic Indexes for Areas [SEIFA] index of disadvantage; mean of 1000 and standard deviation of 100; higher scores represent less disadvantage | 0 (0) |

Abbreviations: BMI, body mass index; HRQoL, health-related quality of life; PedsQL, Pediatric Quality of Life Inventory

Supplementary Table 2. Model specifications for 11 imputation approaches applied to the data from the Longitudinal Study of Australian Children.

|  | Strategy | Method for imputing the outcome variable (HRQoL at wave 3) | Method for imputing the auxiliary variable (HRQoL at wave 1) | MICE or MVNI | Number of variables in the imputation model | Imputations generated? | Error/warning message in Stata (where applicable) |
| --- | --- | --- | --- | --- | --- | --- | --- |
| Original model | Impute all HRQoL items using ordinal logistic regression | Item level, ordinal logistic regression | Item level, ordinal logistic regression | MICE | 54 | No | perfect predictor(s) detected |
| 1 | As original model, plus augmented data | Item level, ordinal logistic regression | Item level, ordinal logistic regression | MICE | 54 | No | regression failed to converge on augmented data |
| 2 | Impute all HRQoL items using linear regression | Item level, linear regression | Item level, linear regression | MICE | 54 | Yes |  |
| 3 | Impute all HRQoL items using PMM | Item level, PMM | Item level, PMM | MICE | 54 | Yes |  |
| 4 | Impute wave 1 HRQoL (auxiliary) as a binary variable | Item level, ordinal logistic regression | Total score level, logistic regression | MICE | 34 | No | perfect predictor(s) detected |
| 5 | Impute wave 1 HRQoL (auxiliary) as a binary variable, plus augmented data | Item level, ordinal logistic regression | Total score level, logistic regression | MICE | 34 | No | logit failed to converge on observed data |
| 6 | Impute wave 3 HRQoL (outcome) as a binary variable | Total score level, logistic regression | Item level, ordinal logistic regression | MICE | 32 | No | perfect predictor(s) detected |
| 7 | Impute wave 3 HRQoL (outcome) as a binary variable, plus augmented data | Total score level, logistic regression | Item level, ordinal logistic regression | MICE | 32 | No | convergence not achieved. logit failed to converge on observed data |
| 8 | Impute HRQoL (outcome and auxiliary) as binary variables | Total score level, logistic regression | Total score level, logistic regression | MICE | 12 | Yes |  |
| 9 | Impute all HRQoL items using MVNI | Item level | Item level | MVNI | 54 | Yes |  |
| 10 | Impute all items (as indicators) using MVNI. To obtain ordinal values for HRQoL items, the HRQoL items are imputed as indicator variables followed by projected-distance based rounding^1^ | Item level, impute indicator variables | Item level, impute indicator variables | MVNI | 186^#^ | No | collinear imputation (dependent) variables detected |
| 11 | Impute HRQoL at the total score level using MVNI. Adapting rounding performed after imputation to obtain the binary outcome variable for the analysis^2^ | Total score level, MVNI | Total score level, MVNI | MVNI | 12 | Yes |  |

Abbreviations: HRQoL, health-related quality of life; MICE, multivariate imputation by chained equations; MVNI, multivariate normal imputation; PMM, predictive mean matching

^#^176 of the variables in this model were indicator variables (i.e. 44 HRQoL items were represented by 4 indicator variables each)

**References**

1. Galati JC, Seaton KA, Lee KJ, Simpson JA, Carlin JB. Rounding non-binary categorical variables following multivariate normal imputation: evaluation of simple methods and implications for practice. Journal of Statistical Computation and Simulation. 2014;84(4):798-811.

2. Bernaards CA, Belin TR, Schafer JL. Robustness of a multivariate normal approximation for imputation of incomplete binary data. Statistics in Medicine. 2007;26(6):1368-82.
